# A case report of SMARCA4-deficient undifferentiated gastric carcinoma diagnosed by endoscopic ultrasound-guided fine needle aspiration

**DOI:** 10.3389/fonc.2026.1763757

**Published:** 2026-04-13

**Authors:** Yuan Chen, Chao Ren, Peifei Shi

**Affiliations:** 1 Department of Gastroenterology, Jinhua Municipal Central Hospital, Jinhua, China; 2 Department of Hepatobiliary and Pancreatic Surgery, Jinhua Municipal Central Hospital, Jinhua, China; 3 Department of Intensive Care Unit, Jinhua Municipal Central Hospital, Jinhua, China

**Keywords:** diagnosis, endoscopic ultrasound-guided fine needle aspiration, malignancy, SMARCA4-deficient, undifferentiated gastric carcinoma

## Abstract

Gastric SMARCA4-deficient undifferentiated carcinoma is an exceptionally rare and highly aggressive malignancy characterized by loss of SMARCA4 expression. Due to its rarity and nonspecific clinical presentation, it poses considerable challenges for diagnosis and management. In this study, we describe the case of a 55-year-old man who was admitted after an incidental finding of a retroperitoneal mass. Contrast-enhanced abdominal computed tomography (CT) and magnetic resonance imaging (MRI) revealed a soft-tissue lesion located in the hepatogastric region. Endoscopic ultrasound (EUS) was subsequently performed to assess the lesion further, enabling precise evaluation of the depth of invasion and facilitating tissue acquisition via EUS-guided fine-needle aspiration (FNA). Cytological examination and immunohistochemical analysis of the EUS-FNA specimen confirmed the diagnosis of SMARCA4-deficient undifferentiated gastric carcinoma. Following disclosure of the pathological diagnosis, the patient declined further treatment and chose to discontinue medical care. He died one month later during follow-up. This case underscores the importance of clinical awareness and prompt diagnostic evaluation, particularly the value of EUS-FNA, in the early identification of SMARCA4-deficient undifferentiated gastric carcinoma, which may ultimately contribute to improved clinical outcomes.

## Introduction

Gastric SMARCA4-deficient undifferentiated carcinoma is an uncommon and highly aggressive subtype of gastric cancer defined by the loss of SMARCA4 gene expression. The SMARCA4 gene encodes Brahma-related gene 1 (BRG1), a key catalytic subunit of the SWItch/Sucrose Non-Fermentable (SWI/SNF) chromatin-remodeling complex, which plays a crucial role in chromatin regulation and the maintenance of cellular homeostasis (1). Disruption of SMARCA4 function has been linked to poorly differentiated, high-grade malignancies and is often associated with unfavorable clinical outcomes.

The occurrence of SMARCA4 deficiency in gastric carcinoma is extremely rare, with an estimated incidence of approximately 1.8% based on SMARCA4 expression analysis in a cohort of 2,000 gastrointestinal cancer cases (2). Most patients with SMARCA4-deficient gastric carcinoma present at an advanced stage and frequently show nonspecific symptoms, including dysphagia, abdominal discomfort, or an abdominal mass (3). Histopathological examination typically demonstrates sheets of poorly differentiated or undifferentiated tumor cells that often lack distinctive morphological characteristics and show limited or absent expression of conventional diagnostic biomarkers, making accurate diagnosis particularly challenging (4).

Therefore, advanced tissue-acquisition techniques such as endoscopic ultrasound–guided fine-needle aspiration (EUS-FNA) have become increasingly valuable. EUS-FNA enables precise sampling of submucosal and extramural lesions, providing sufficient tissue for immunohistochemical analysis and supporting a definitive diagnosis when routine endoscopic biopsies yield inconclusive results (5). This case report highlights the diagnostic utility of EUS-FNA. Furthermore, it underscores the need for greater clinical awareness of SMARCA4-deficient undifferentiated gastric carcinoma, a rare but highly aggressive entity that remains underrecognized in routine clinical practice. This case report emphasizes the diagnostic value of EUS-FNA. It highlights the importance of increasing clinical awareness of SMARCA4-deficient undifferentiated gastric carcinoma, a rare yet highly aggressive tumor that remains underrecognized in routine clinical practice.

## Case presentation

A 55-year-old man was admitted after a retroperitoneal mass had been detected approximately two weeks earlier. The abnormality was initially identified during evaluation prompted by a positive fecal occult blood test obtained through a community colorectal cancer screening program. Further gastrointestinal endoscopy revealed a shallow mucosal depression located 35 cm from the incisors in the esophagus. Histopathological examination of the biopsy specimen demonstrated chronic atrophic gastritis with *Helicobacter pylori* infection and multiple gastric polyps. Colonoscopy was also performed, during which several polyps were removed. The patient was later hospitalized at a local institution for further assessment of the esophageal lesion. A repeat endoscopy on July 22, 2024, showed an irregular mucosal lesion in the mid-esophagus, and pathological analysis confirmed high-grade intraepithelial neoplasia.

Laboratory investigations at admission revealed a platelet count of 371×10^9^/L, hypersensitive C-reactive protein of 16.23 mg/L, and carcinoembryonic antigen of 6.96 ng/mL. Liver function tests demonstrated elevated gamma-glutamyl transferase (80.7 U/L) and lactate dehydrogenase (604 U/L), while renal function remained within normal limits. Electrocardiography showed sinus rhythm with evidence of left ventricular hypertrophy. Chest computed tomography (CT) revealed a small nodule in the right upper lobe, enlarged lymph nodes in the posterior mediastinum, and scattered fibrotic foci in both lungs. The hepatogastric mass persisted, and the gastroesophageal junction appeared indistinct.

**Figure 1:**
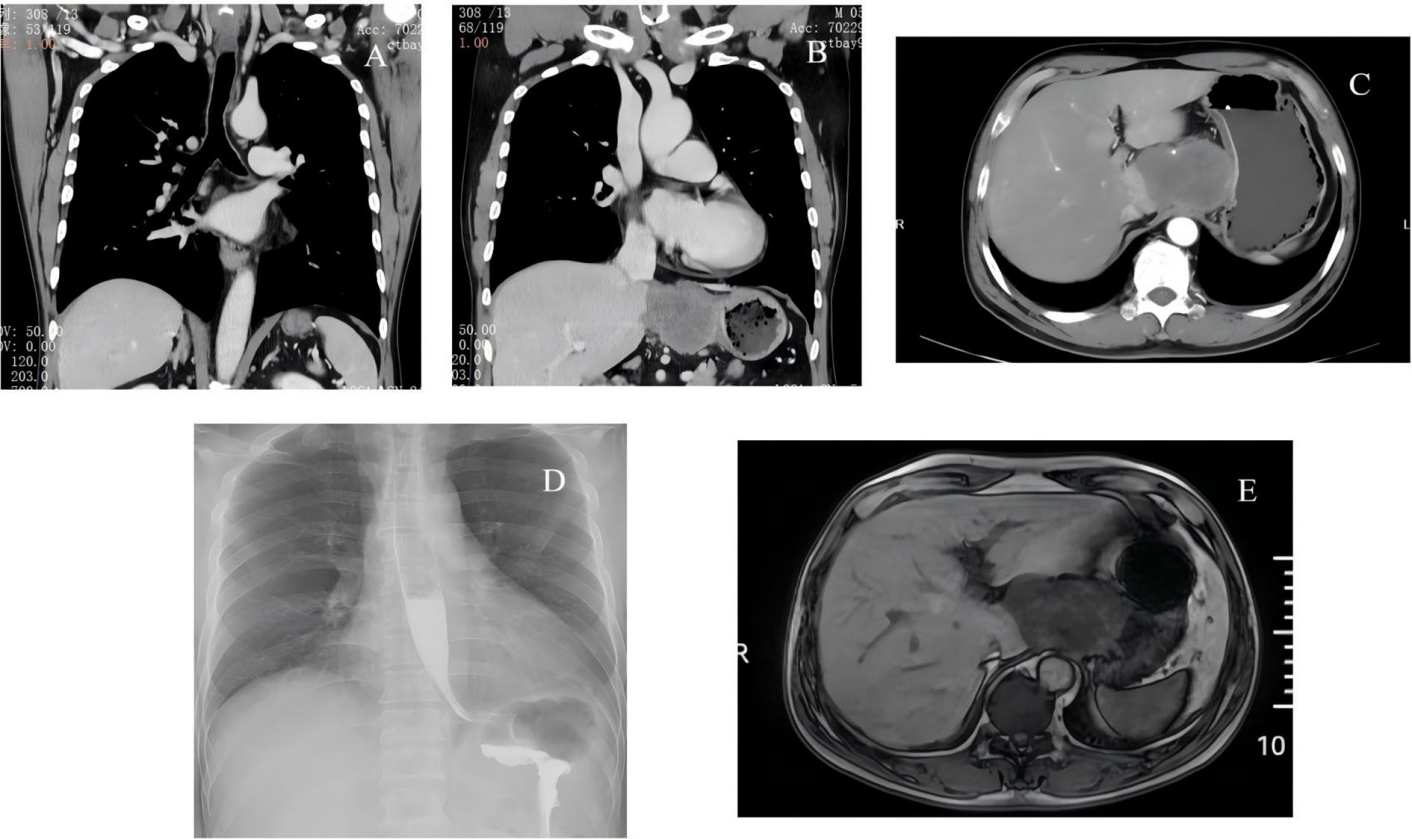
Imaging findings. **(A–C)** Contrast-enhanced computed tomography (CT) images demonstrating a soft-tissue mass located in the hepatogastric space. **(D)** Upper gastrointestinal contrast study showing external compression at the gastric outlet. **(E)** Magnetic resonance imaging (MRI) demonstrates a well-defined mass in the hepatogastric region.

An enhanced abdominal CT scan demonstrated a soft-tissue mass within the hepatogastric space that encased the celiac trunk and branches of the hepatic artery, with poorly defined margins adjacent to the liver. These findings raised suspicion for lymphoma or a possible extramural tumor originating from the caudate lobe of the liver. Subsequent magnetic resonance imaging (MRI) identified a mass in the hepatogastric region with imaging characteristics suggestive of lymphoid hyperplasia or paraganglioma (**Figure 1**). Because the diagnosis remained uncertain, the patient was transferred to our general surgery department on July 27, 2024, for further evaluation and management.

The patient had a known history of hypertension that was well controlled with long-term medication. On admission, his vital signs were as follows: pulse 100 beats per minute, respiratory rate 20 breaths per minute, blood pressure 141/91 mmHg, and body temperature 36.9 °C. He was conscious and oriented, without jaundice of the skin or sclera, and no superficial lymphadenopathy was detected. Cardiopulmonary examination revealed no abnormalities. The abdomen was soft and non-tender, with no palpable masses, and bowel sounds were present at approximately four times per minute. No edema was observed in the lower extremities.

**Figure 2:**
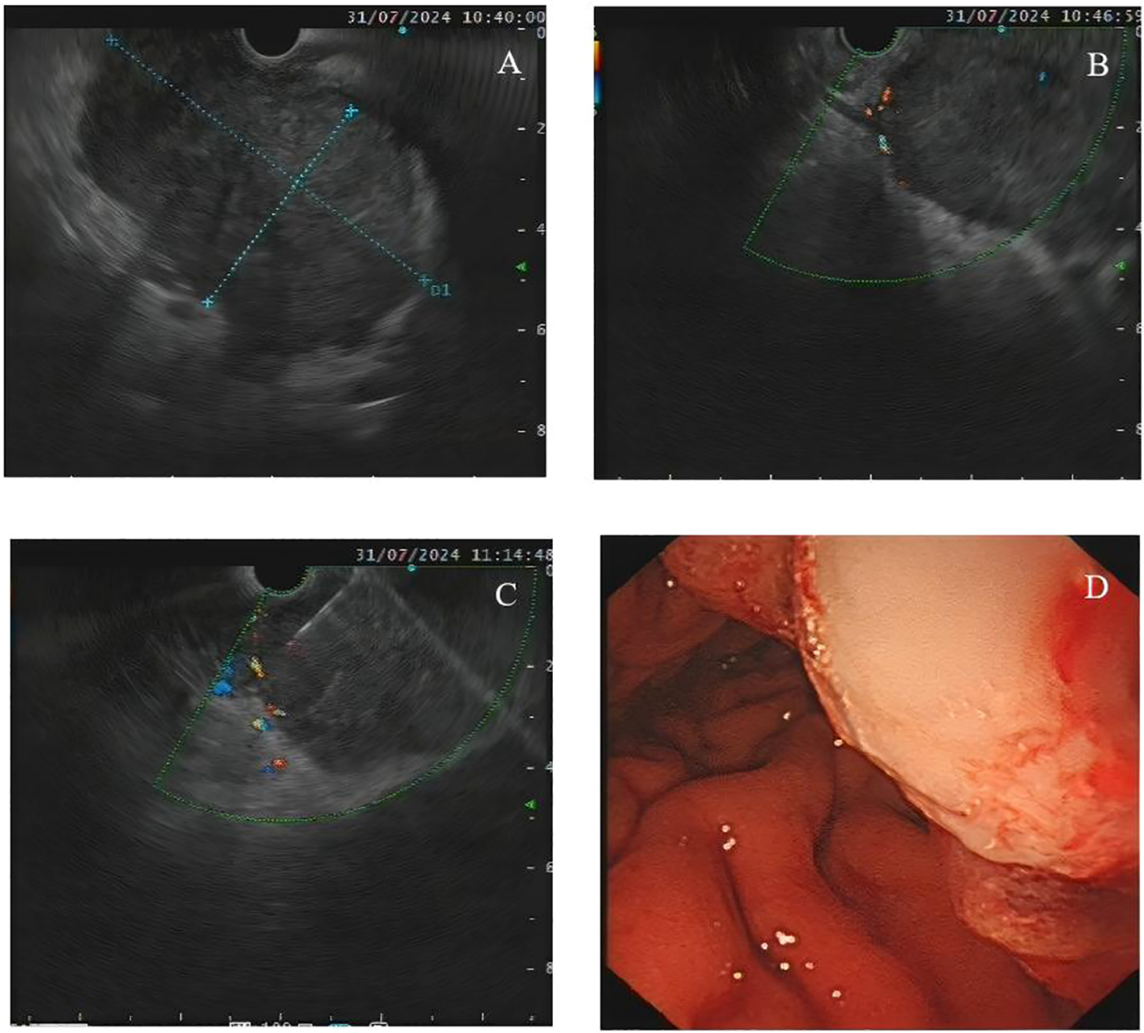
Endoscopic ultrasound–guided fine-needle aspiration (EUS-FNA). **(A)** EUS image demonstrating a large hypoechoic mass located between the liver and stomach. **(B)** The lesion appeared to originate from the deep parenchymal (basal) layer, raising suspicion for a stromal tumor. **(C)** Real-time EUS-guided needle puncture of the lesion was performed using a 19-gauge needle. **(D)** Endoscopic view after tissue sampling showing mild bleeding on the tumor surface.

During hospitalization, the patient developed progressively worsening dysphagia and was eventually able to tolerate only liquid intake. An upper gastrointestinal contrast study performed on July 30, 2024, showed gastric outlet obstruction due to external compression. On July 31, 2024, endoscopic ultrasound (EUS) revealed a mass within the hepatogastric space that appeared to originate from the gastric wall, raising suspicion for a gastric stromal tumor in the setting of early esophageal cancer (**Figure 2**). Under EUS guidance, fine-needle aspiration was performed using a 19-gauge Cook endoscopic needle, obtaining multiple tissue cores for pathological examination (**Figure 2**).

**Figure 3:**
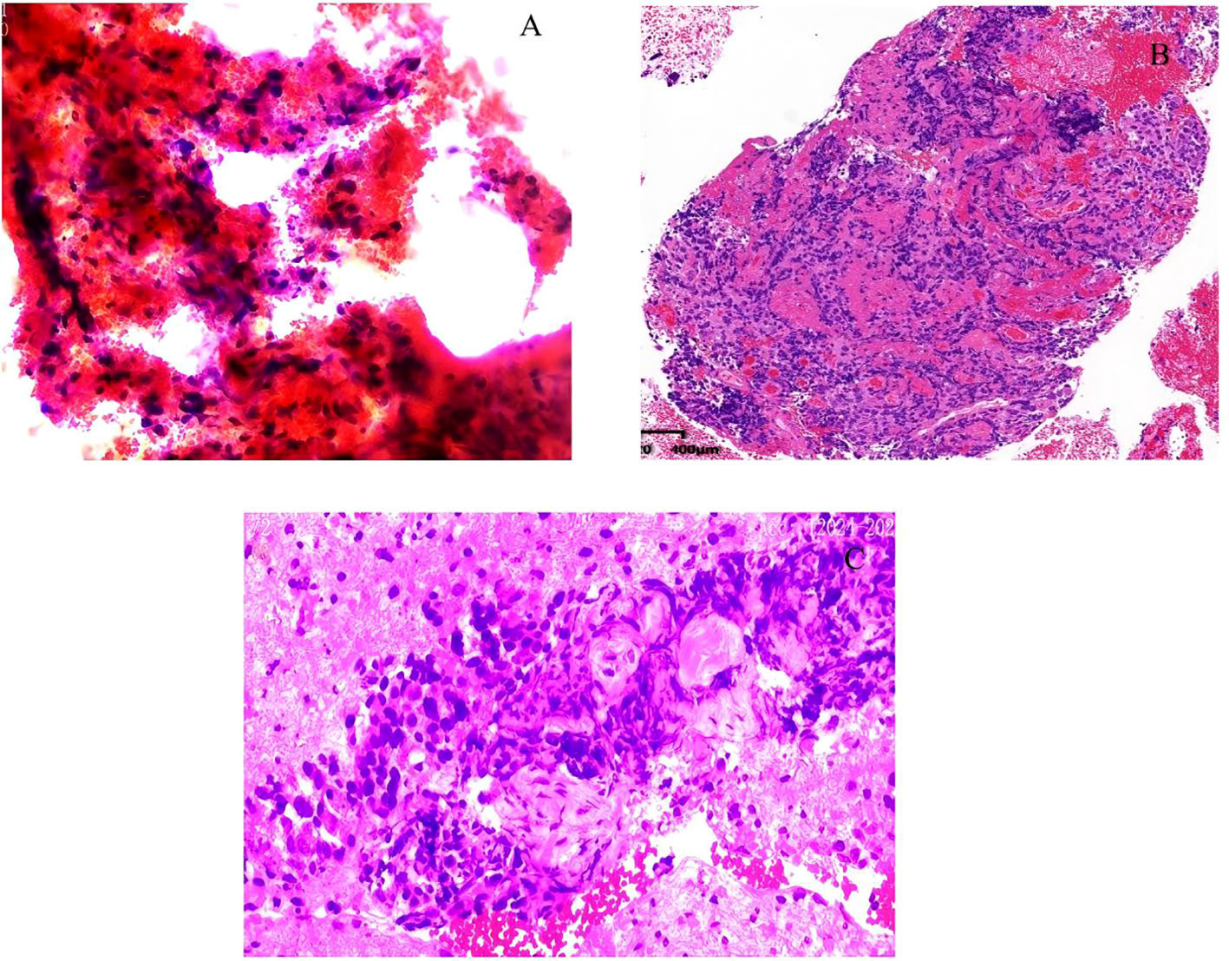
Cytological and histopathological findings from endoscopic ultrasound–guided fine-needle aspiration (EUS-FNA). **(A)** Cytological smear showing scattered atypical cells. **(B)** Hematoxylin and eosin (H&E) staining (×100 magnification) demonstrating features consistent with a highly malignant neoplasm. **(C)** Liquid-based cytology reveals malignant epithelial cells.

**Figure 4:**
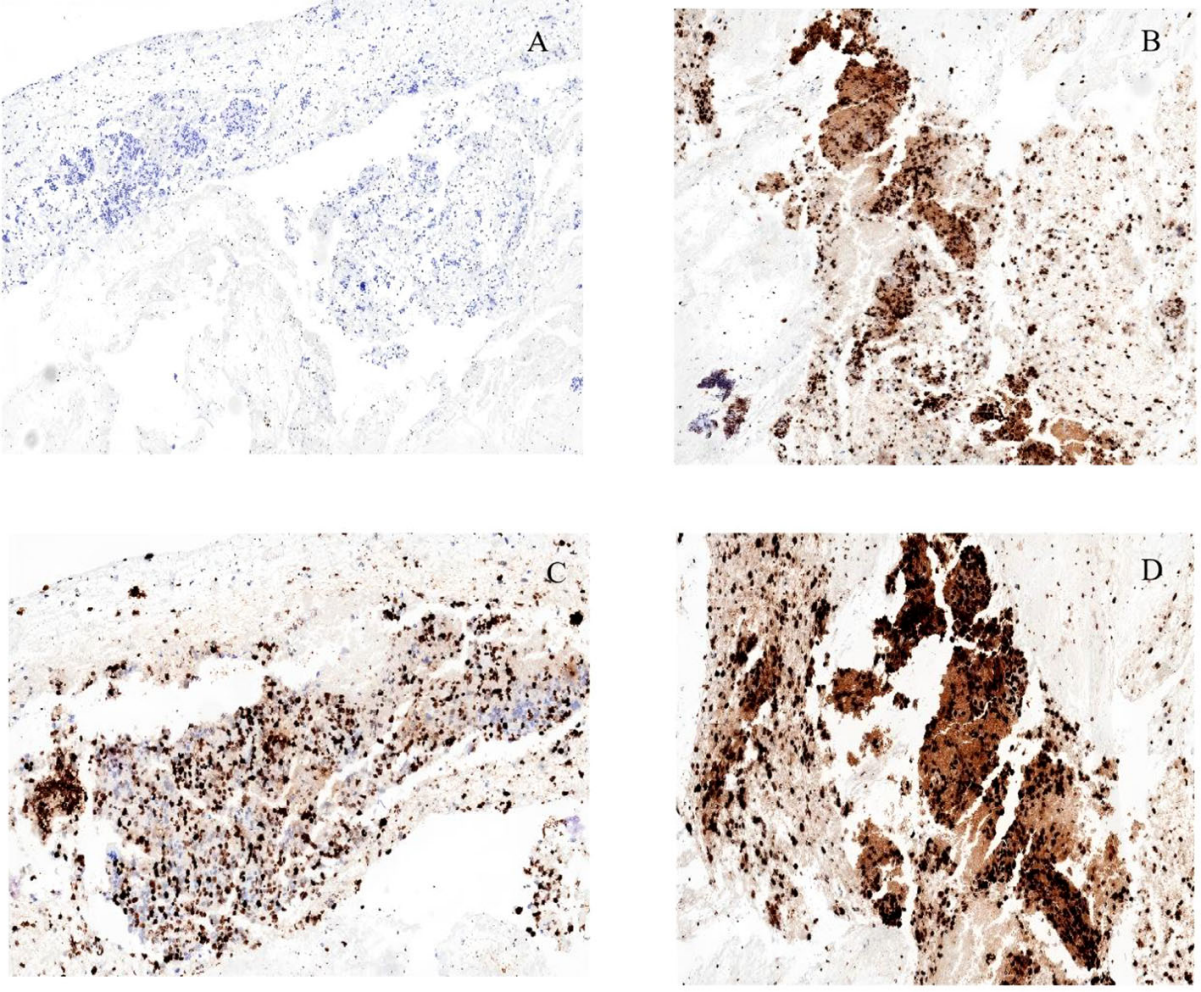
Immunohistochemical characteristics of the tumor. **(A)** Complete loss of SMARCA4 expression, confirming SMARCA4 deficiency. **(B)** Positive nuclear staining for mutant p53. **(C)** High proliferative activity demonstrated by Ki-67 positivity (approximately 60%). **(D)** Preserved expression of INI-1.

Cytological smears demonstrated scattered atypical cells accompanied by areas of necrosis, consistent with a malignant process (**Figure 3**). Histopathological evaluation of the biopsy specimen revealed a highly aggressive neoplasm consistent with undifferentiated carcinoma, characterized by loss of the SWI/SNF chromatin-remodeling complex component SMARCA4 (**Figure 4**). Liquid-based cytology further confirmed the presence of malignant cells. Immunohistochemical analysis supported the diagnosis of poorly differentiated carcinoma, with positive staining for Ki-67, INI-1, and P53, and negative staining for CK7, CK5/6, CD117, and other lineage-associated markers. SMARCA4 expression was completely absent, which represented a key diagnostic feature (**Figures 3**, **4**).

At the referring institution, the patient had initially been diagnosed with esophageal carcinoma accompanied by a gastric mass, with pathological confirmation of the esophageal lesion. However, the origin and nature of the gastric mass remained uncertain, complicating treatment planning. Following transfer to our hospital, EUS-FNA of the gastric lesion enabled a definitive diagnosis of SMARCA4-deficient undifferentiated carcinoma, a rare and highly aggressive tumor. After receiving the pathological diagnosis and considering his financial circumstances, the patient chose to decline further treatment and was discharged. He died one month later during follow-up.

## Discussion

The present case of SMARCA4-deficient undifferentiated carcinoma illustrates the substantial diagnostic challenges posed by this rare and highly aggressive malignancy. Previous reports have described a considerable risk of misdiagnosis, largely due to the tumor’s atypical morphology and nonspecific imaging findings, which frequently lead clinicians to consider alternative diagnoses such as lymphoma or gastrointestinal stromal tumors (6, 7). Therefore, accurate identification relies heavily on immunohistochemical evaluation, particularly the demonstration of absent SMARCA4 expression, which has consistently been recognized as a key diagnostic feature distinguishing this carcinoma from other undifferentiated neoplasms (7–9). Consistent with earlier studies, SMARCA4-deficient tumors demonstrate an extremely aggressive clinical course characterized by rapid progression and poor prognosis. These observations highlight the importance of increasing clinical awareness, promoting early diagnostic evaluation, and advancing research to develop effective targeted therapies for this rare but lethal disease.

According to the current literature and routine clinical practice, no standardized treatment guidelines have been specifically established for SMARCA4-deficient undifferentiated gastric carcinoma (**Table 1**). As a result, therapeutic strategies generally follow treatment principles for advanced gastric cancer while also considering the unique biological characteristics associated with SMARCA4 deficiency. For resectable disease, radical gastrectomy with D2 lymphadenectomy remains the primary treatment approach. Perioperative or adjuvant chemotherapy regimens based on fluoropyrimidine combined with platinum are commonly recommended (8, 9). In cases of unresectable, locally advanced, or metastatic disease, first-line therapy typically involves a combination of PD-1/PD-L1 inhibitors with fluoropyrimidine- and platinum-based chemotherapy, given the relatively high immunogenicity and frequent PD-L1 expression observed in SMARCA4-deficient tumors. For patients with refractory or recurrent disease, second- or later-line treatment options may include paclitaxel, irinotecan, or participation in clinical trials targeting deficiencies in the SWI/SNF chromatin-remodeling complex (10, 11).

**Table 1:** Clinical and pathological characteristics reported in SMARCA4-deficient gastric carcinoma.

No.	Reference	Age/Gender	Tumor location	EUS Findings	IHC/Molecular findings	Clinical stage	Treatment	Outcome
1	Wu JY (2)	49-72/M (6 cases), 70-72/F (2 cases)	GEJ (3 cases), gastric body/antrum (4 cases), fundus (1 case)	NA	IHC: SMARCA4 loss, INI1 (SMARCB1) diffuse strong positive, Ki-67 70%-90% no NGS data	IV (4 cases), III (2 cases), unknown (2 cases)	Radical gastrectomy (3 cases), chemoimmunotherapy /radiotherapy (3 cases) unknown (2 cases)	Median OS = 10 months; 2 cases died within 3 months
2	Lin JL (3)	59/M	Gastric body	A mass measuring approximately 7 cm with blackish pigmentation	IHC: SMARCA4 loss, INI1 intact; no NGS data	Unknown	Radical gastrectomy and lymph node dissection	NA
3	Misra S (4)	28/M	Gastric cardia	Undifferentiated carcinoma cells with focal squamous differentiation; discohesive; high mitotic activity	IHC: SMARCA4 loss, p63 (+), p40 (+), INI1 preserved; NGS: SMARCA4 frameshift mutation	IV (liver and lymph node metastasis)	Surgical resection and FOLFOX chemotherapy, immunotherapy (nivolumab)	OS = 24 months
4	An HR (5)	21–67/M (6 cases)	Gastric antrum (2 case), body (2 cases), cardia (2 case)	2 cases with ulcerofungating, 3 cases with ulceroinfiltrative and 1 case with ulcerative	IHC: SMARCA4 loss, focal weak positivity for cytokeratin AE1/AE3 or EMA; NGS: SMARCA4 gene mutations (5 cases)	IV (5 cases), III (1 case)	Palliative chemotherapy/ immunotherapy(EP(2 cases),Nivolumab +XELOX(2 cases),Adjuvant TS-1 (1 case)and FOLFOX (1 case))	Median OS = 7 months; 2 cases died within 3 months
5	Zhong Y (7)	57-76/M (4 cases)	Gastric antrum (2 cases), body (1 case), cardia (1 case)	Undifferentiated cells; focal necrosis and rhabdoid features (2 cases)	IHC: SMARCA4 loss, INI1 retained, positivity for epithelial markers (AE1/AE3 or EMA); No NGS data	III (1 case), IV (2 cases) unknown (1 case)	Adjuvant chemotherapy + surgery (3 cases), palliative chemoimmunotherapy (1 case)	Median OS = 7 months; 1 case unknown
6	Cheng XL (10)	69/M, 61/M (2 cases)	Gastric fundus and cardia (2cases)	Large ulcerated mass diffusely infiltrated lamellar growth	IHC: SMARCA4 loss, INI1 intact, Ki-67 (≧80%, PD-L1 CPS 5/10; No NGS data	IV(Case 1), II (Case 2)	Radical gastrectomy + chemotherapy (EP/paclitaxel oxaliplatin)+ anti-PD1 immunotherapy(sintilimab)	OS=18months (Case 1) and 3 months (Case 2), respectively
7	Chen L (11)	50/F	Gastric body	The lesion exhibited diffuse infiltration to the entire layer of the stomach wall	IHC: SMARCA4 loss, INI1 intact, AE1/AE3 (+), CK8/18 (+), Ki-67 60%; NGS: SMARCA4 frameshift mutation	Advanced (stage unspecified)	Radical resection; chemotherapy unspecified	Outcome not specified
8	Kim SH (12)	80/M, 71/F	Gastric cardia (2 cases)	A large ulcerofungating mass without evidence of glandular or squamous differentiation	IHC: SMARCA4 loss, High Ki-67 No NGS data	IV (2 cases)	Total gastrectomy+ chemotherapy (Paclitaxel + Carboplatin/XELOX )	Transferred for hospice care(1 case) and surveillance without recurrence (1 case)

EUS, Endoscopic Ultrasound; IHC, Immunohistochemistry; NGS, Next-Generation Sequencing; NA, Unknown; FOLFOX, fluorouracil, leucovorin, and oxaliplati; EP, etoposide and cisplatin, TS-1, tegafur, gimeracil, and oteracil potassium, XELOX, capecitabine and oxaliplatin; OS, Overall Survival; GEJ, Gastroesophageal Junction; CKpan, Pan-Cytokeratin; EMA, epithelial membrane antigen;CPS, Combined Positive Score.

Previous studies have also emphasized that the atypical clinical and pathological characteristics of SMARCA4-deficient tumors often contribute to diagnostic confusion, with initial diagnoses frequently favoring entities such as gastrointestinal stromal tumors or lymphoma (7, 12). These diagnostic complexities highlight the necessity of comprehensive immunohistochemical analysis, particularly assessment of SMARCA4 expression, which remains essential for establishing an accurate diagnosis (13). In a retrospective analysis by Horton et al., which examined 14 cases of undifferentiated carcinoma at the gastroesophageal junction with SMARCA4 or SMARCA2 deletions, immunohistochemical evaluation demonstrated loss of SMARCA4 expression in 85.7% of cases and loss of SMARCA2 in 50%, underscoring the important role of these genes in tumor development (14). Concurrent alterations involving SMARCA4 and SMARCA2, together with abnormal p53 expression, suggest that these molecular abnormalities contribute significantly to the pathogenesis of undifferentiated carcinoma. In the present case, immunohistochemical analysis demonstrated complete loss of SMARCA4 expression, along with positive staining for p53 and Ki-67, findings that are consistent with patterns reported in the literature.

Moreover, the EUS combines endoscopic visualization with high-frequency ultrasonography, enabling high-resolution imaging of the gastrointestinal tract and surrounding structures (15). The technique employs an ultrasound transducer mounted at the distal end of the endoscope, which is inserted into the gastric lumen to generate detailed cross-sectional images. EUS can clearly delineate the five layers of the gastric wall: superficial mucosa, deep mucosa, submucosa, muscularis propria, and serosa (16). This layered visualization is essential for accurately assessing the depth of tumor invasion, a key prognostic factor in gastric malignancies, including rare entities such as SMARCA4-deficient undifferentiated carcinoma and rhabdoid-like carcinoma of the stomach. In these aggressive tumors, extensive infiltration across multiple layers of the gastric wall is frequently observed.In comparison, conventional endoscopy provides excellent visualization of the mucosal surface. Moreover, it lacks the capacity to assess deeper layers, such as the submucosa and muscularis propria, limiting its ability to fully evaluate tumour invasion (17, 18). Therefore, the superior depth resolution of EUS makes it particularly valuable for early detection, staging, and clinical decision-making in rare and infiltrative gastric malignancies.

Another advantage of EUS lies in its ability to detect early-stage gastric lesions, particularly those arising from the submucosal or muscularis propria layers, which may remain subtle or entirely undetectable on conventional imaging modalities (18, 19). Owing to its high-resolution ultrasound capabilities, EUS can identify subtle structural abnormalities in the gastric wall, including hypoechoic areas or irregular echo patterns that may represent early neoplastic infiltration but are often overlooked by CT, MRI, or routine endoscopy (20, 21). This capability is particularly valuable for rare gastric malignancies, such as SMARCA4-deficient carcinoma, which is typically poorly differentiated and may initially proliferate in the submucosa, making early detection difficult with endoscopy alone. Furthermore, EUS facilitates targeted tissue acquisition through EUS-guided FNA, allowing precise sampling of submucosal and extramural lesions for cytological, histopathological, and immunohistochemical evaluation (17, 22–24). This approach is critical for confirming SMARCA4 deficiency and distinguishing these tumors from other gastric neoplasms, supporting accurate differential diagnosis and appropriate clinical management.

Compared with CT and MRI, EUS provides substantially higher resolution for evaluating the layered structure of the gastric wall, enabling more precise assessment of tumor invasion depth and local staging (15). Although CT and MRI remain valuable imaging modalities for detecting distant metastases and evaluating overall tumor burden, they lack the spatial resolution required to identify early-stage or submucosal lesions, which is particularly important in rare malignancies such as SMARCA4-deficient gastric carcinoma (25, 26). In comparison, EUS enables detailed visualization of subtle abnormalities within the mucosal, submucosal, and muscular layers of the stomach. When combined with EUS-FNA, it enables accurate tissue sampling, facilitating histopathological and molecular differentiation of SMARCA4-deficient gastric carcinoma from other tumor types. Further, advanced EUS techniques such as elastography and contrast-enhanced EUS can assess tissue stiffness, vascularity, and perfusion, providing further information regarding tumor aggressiveness and depth of invasion (27). Given these advantages, EUS with tissue acquisition is widely considered a key diagnostic modality for evaluating rare gastric cancers that require detailed molecular characterization, including confirmation of SMARCA4 deficiency, to guide prognosis and therapeutic decision-making.

Several limitations of this study should also be acknowledged. Although SMARCA4 loss was confirmed by immunohistochemistry, advanced genomic analyses, including whole-exome sequencing, RNA sequencing, and methylation profiling, were not performed, limiting deeper molecular characterization. Further, EUS-FNA yields relatively limited tissue, which may not fully capture tumor heterogeneity or detect additional coexisting molecular alterations. Furthermore, other emerging biomarkers, such as SMARCA2 status, PD-L1 expression, mismatch repair proteins, and tumor mutational burden, were not evaluated, which may restrict comprehensive prognostic assessment.

In summary, SMARCA4-deficient undifferentiated gastric carcinoma represents a rare but exceptionally aggressive form of gastric malignancy characterized by distinctive molecular alterations, complete loss of SMARCA4 expression, and significantly undifferentiated histological features. These tumors are frequently diagnosed at advanced stages because of their subtle early manifestations and rapid infiltrative growth. Clinical management remains challenging owing to diagnostic uncertainty, limited responsiveness to conventional chemotherapy, and the absence of well-defined treatment guidelines. High-resolution EUS has emerged as a critical diagnostic tool in suspected cases, offering detailed visualization of the gastric wall layers and enabling accurate assessment of tumor invasion depth. When combined with EUS-guided FNA, this technique allows acquisition of high-quality tissue samples necessary for immunohistochemical and molecular analyses, particularly for confirming SMARCA4 deficiency and differentiating these tumors from other undifferentiated or submucosal lesions. This case provides additional clinical insight into the presentation, diagnostic complexity, and aggressive biological behaviour of SMARCA4-deficient undifferentiated carcinoma, while also underscoring the need for greater clinical awareness and further research. Advancements in molecular diagnostics, earlier detection strategies, and the development of targeted therapies will be essential for improving diagnostic accuracy, enabling personalized treatment approaches, and ultimately enhancing survival outcomes for patients affected by this devastating disease.

## Data Availability

The raw data supporting the conclusions of this article will be made available by the authors, without undue reservation.
